# A Neutrophil-like Cell Model as Substitute for Human Neutrophils in NETs and Thrombosis Research

**DOI:** 10.3390/cells15060541

**Published:** 2026-03-18

**Authors:** Yu Shi, Helen R. McPherson, Timea Feller, Simon D. A. Connell, Helen Philippou, Robert A. S. Ariëns, Julia S. Gauer

**Affiliations:** 1 Discovery and Translational Science Department, Leeds Institute of Cardiovascular and Metabolic Medicine, University of Leeds, Leeds LS2 3AA, UK; yu.shi@ki.se (Y.S.); h.mcpherson@leeds.ac.uk (H.R.M.); t.feller@leeds.ac.uk (T.F.); h.philippou@leeds.ac.uk (H.P.); r.a.s.ariens@leeds.ac.uk (R.A.S.A.); 2 Department of Medicine, Huddinge, Karolinska Institutet, 141 52 Huddinge, Sweden; 3 Sir William Henry Bragg Building, School of Physics and Astronomy, University of Leeds, Leeds LS2 9JT, UK; s.d.a.connell@leeds.ac.uk

**Keywords:** neutrophil extracellular traps (NETs), PLB-985 cells, fibrin fibre, scanning electron microscopy (SEM), atomic force microscopy (AFM), in vitro neutrophil-like model

## Abstract

Neutrophil extracellular traps (NETs) critically influence thrombosis by promoting platelet aggregation, fibrin formation, and thrombus stabilisation. However, primary human neutrophils present experimental limitations, including short lifespan ex vivo and ethical concerns. In this article, we discuss the available data on PLB-985 cells, a neutrophil-like model with potential to replace human neutrophils in research. Additionally, we present novel structural comparisons showing that both PLB-985- and human neutrophil-derived NETs significantly increased fibrin fibre thickness compared to thrombin-only controls, with similar fibre morphology across conditions. Notably, we also see spherical particles resembling microvesicles within PLB-985-derived NETs, suggesting potential additional procoagulant effects via microvesicle-associated tissue factor level in these cells. New and existing data presented in this article suggest that differentiated PLB-985 cells are able to effectively replicate key structural and functional aspects of human neutrophil NETs. These observations support the use of PLB-985 cells as an ethical, reproducible, and practical alternative for in vitro studies of NETs. Further characterisation is required to determine differences between human neutrophils and neutrophil-like models in macrovesicle formation and implication in NET-related thrombosis research.

## 1. Introduction

Neutrophils, generated from the myeloid lineage in the bone marrow, are the most abundant leukocytes in blood and constitute the first line of defence in innate inflammation, efficiently capturing and killing bacteria and other pathogens [1]. Their principal antimicrobial functions include phagocytosis, degranulation, generation of reactive oxygen species (ROS), and the formation of neutrophil extracellular traps (NETs) [2]. Recently, neutrophils have been implicated in the crosstalk between innate immunity and haemostasis [3]. However, NET research is constrained by the short ex vivo lifespan of primary neutrophils and donor variability, motivating the development of reproducible neutrophil-like cell models. This article will explore the suitability of such models to replace human neutrophils in thrombosis research and beyond by discussing structural comparison data between primary and neutrophil-like cells in the context of the current available literature.

### 1.1. Neutrophils and NETosis

Neutrophils, which are generated from the myeloid cell lineage in the bone marrow, are the most abundant leukocyte in the blood. Neutrophils act as the first line of defence in innate inflammatory responses, effectively catching and killing bacteria and pathogens [1]. The main antimicrobial functions of these cells are phagocytosis, degranulation, and generation of reactive oxygen species (ROS) [2]. Another vital process involved in neutrophils’ role in the innate immune response is their ability to produce neutrophil extracellular traps (NETs). The process of NET formation, termed NETosis, occurs when activated neutrophils extrude a modified form of chromatin, decorated with cytoplasmic granules, histones, and granular proteins [4]. During NETosis, histones are citrullinated to induce decondensation of chromatin, followed by decoration of chromatin with other components inside the neutrophil when the nuclear membrane disintegrates [5]. This process induces the rupture of the cytoplasmic membrane and the release of NETs, which structurally appear as a fibrous network [4]. NETosis occurs when neutrophils are activated by triggers such as microbial and inflammatory stimuli (e.g., bacteria and fungi) or by Phorbol 12-myristate 13-acetate (PMA), which is often used in laboratory experiments [3,6]. Fuchs et al. showed that NETosis is a form of active programmed cell death, favoured at ≤2% fetal calf serum concentrations, leading to unique morphological changes in the cell’s nucleus, such as disintegration of the nuclear membrane [7]. They also showed that NETosis depends on nicotinamide adenine dinucleotide phosphate (NADPH) oxidase-derived hydrogen peroxide (H_2_O_2_) but is caspase-independent. As caspase plays a critical role in apoptosis, this distinguishes the process of NETosis from apoptosis/necrosis where the cell’s nuclear membrane remains intact [7]. Furthermore, NETosis has previously been classified into two categories, namely, suicidal NETosis and vital NETosis. The process termed suicidal NETosis is normally slower and mostly stimulated by chemical stimuli (e.g., PMA), while vital NETosis has been shown to occur more rapidly and via stimulation by microbial-specific stimuli (e.g., lipopolysaccharides of Gram-negative bacteria) [8]. A key distinction between these the two forms of NETosis is that during vital NETosis the functions of the cell remain active, for the NET formation occurs independent of cellular suicide [8,9].

### 1.2. Neutrophils and NETs in Blood Clot Formation

Haemostasis is the physiological process that occurs following injury to the blood vessel that leads to blood clot formation [10]. The process of haemostasis is often broken down into four steps, namely, vasoconstriction, formation of the platelet plugs, activation of the coagulation pathways, and the formation of fibrin [11]. Immediately following injury, the blood vessel walls contract to minimize blood flow to the damage site [12]. Inert circulating platelets are then activated via collagen exposed by the endothelium and aggregate at the site of injury to form a platelet plug [13]. Concurrently, activation of the coagulation cascade leads to the conversion of fibrinogen to fibrin, forming an insoluble clot [14]. Haemostasis is a dynamic process that balances pro- and anticoagulant mechanisms, with dysregulations leading to bleeding or potentially life-threatening blood clots [15].

**Figure 1:**
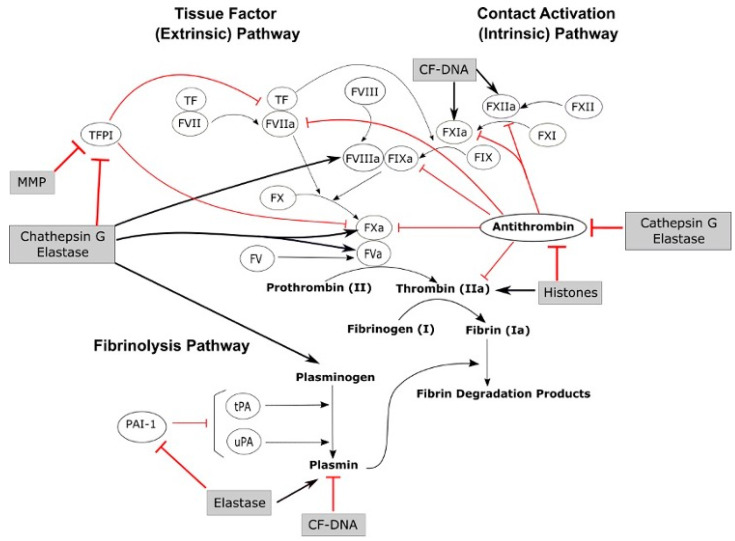
The role of neutrophil-released mediators in the coagulation pathways. Black arrows denote stimulation and activation, red blunt arrows denote inhibition. Neutrophil mediators matrix metalloproteinases (MMPs), cell-free DNA (CF-DNA), histones, cathepsin G, and elastase (grey boxes) influence the coagulation pathways by directly activating procoagulant and fibrinolytic factors, and inhibit the activity of anticoagulants. TFPI: tissue factor pathway inhibitor, tPA: tissue plasminogen activator, uPA: urokinase plasminogen activator, and PAI: plasminogen activator inhibitor [16].

Previous studies have shown that neutrophils play a pivotal role in the crosstalk between innate immunity and haemostasis. For instance, studies have shown that mediators released by neutrophils, such as matrix metalloproteinases (MMPs), cell-free DNA (CF-DNA), histones, cathepsin G, and elastase, can activate coagulation factors (e.g., FXII, FXI, FVIII, FX, FV, and thrombin), inhibit anticoagulant factors (e.g., tissue factor pathway inhibitor (TFPI) and antithrombin), and influence the clot dissolution (fibrinolysis) pathway [16,17] (Figure 1). Nevertheless, the mechanisms through which neutrophils influence coagulation are likely complex. For example, procoagulant effects such as inactivation of TFPI and antithrombin have been attributed to neutrophil elastase, which has conversely been associated with anticoagulant effects through cleavage of fibrinogen [18,19,20]. Recently, neutrophils have been shown to promote clotting in plasma, independently of thrombin, FXII, and FVII [3], suggesting a key role in blood clot formation but with associated mechanisms to be uncovered. The expression of tissue factor (TF), a key initiator of clotting, in neutrophils remains a subject of ongoing debate in the literature. Several studies performed on different animal models indicate that neutrophils are able to express TF, including in human blood [21,22,23,24,25]. Nevertheless, other studies suggest that neutrophils do not express TF themselves but obtain it from monocytes instead through cell–cell interactions [26,27,28]. Therefore, further studies are required to better understand the role of neutrophils in coagulation.

The effect of NETs on haemostasis and cardiovascular disease pathology has also been a topic of interest [29,30]. NETs have been shown to contribute to venous and arterial thrombosis, including venous thromboembolism and stroke, where their presence within thrombi has been tied to resistance to thrombolysis [31,32,33]. For this reason, recent interest in the potential of targeting NETs to reduce or protect against thrombosis has emerged [34,35]. Attention has also recently turned to the potential regulatory effects of drugs frequently used in the treatment of cardiovascular diseases on NET formation. For instance, a recent study on coronary artery disease patients reported a reduction in NET-related proteins following treatment with high-dose statin [36]. One of the suggested mechanisms by which NETs are thought to promote blood clotting is via the activation of the intrinsic pathway of coagulation, for NETs contain DNA that is negatively charged [37]. A recent study using samples from human volunteers showed that NETs promoted clotting independently of thrombin, FXII, FVII, and FXI, producing denser clots with increased resistance to fibrinolysis [3]. In contrast, a study by Von Bruhl et al. found that NETs contributed to deep vein thrombosis formation independent of FXI, but required FXII, in mice models [37]. It has also been shown that NETs are decorated with TF, suggesting that they could also initiate clotting via the extrinsic pathway [37,38]. Another study showed that purified components of NETs, but not intact NETs themselves, were able to promote clotting due to the interactions between histones and DNA [39]. These data highlight the need for further investigations on the mechanisms by which NETs and their components influence coagulation, and the potential modulatory role of anticoagulation agents.

Beyond coagulation, NETs have also been shown to promote clotting through platelet activation. A previous study showed that NETs provided a scaffold for platelet activation, thereby promoting platelet activation and aggregation and initiating clotting independently of fibrin [40]. Furthermore, citrullinated histone H3 (citH3), a biomarker of NETs, has been shown to bind to von Willebrand factor and contribute to the formation of platelet-rich deep vein thrombosis in mice [31]. Some studies showed that platelets can be activated by NETs, which in turn can promote NET formation via a positive feedback loop [41,42,43]. These observations are in agreement with another study showing an interaction between NETs and platelets during NET-induced clotting [44]. It has been suggested that von Willebrand factor, shown to bind to NETs, also interacts with GPIbα in the GPIb-IX-V complex and integrin αIIbβ3 on the platelets, and thus may act as a bridge in the interactions between the two cell types [31,45]. In addition to interacting with NETs, platelets also contribute to the recruitment of neutrophils. This interaction occurs mainly via P-selectin on platelets interacting with P-selectin glycoprotein ligand 1 (PSGL-1) on neutrophils [46,47], but also through other mechanisms, such as platelet GPIbα binding to neutrophil MAC-1, platelet ICAM-2 binding to neutrophil LFA-1, and platelet αIIbβ3 indirectly binding to neutrophil MAC-1 via fibrinogen [48,49]. A recent study demonstrated that dual antiplatelet therapy reduced NET formation in a cancer murine model [50], highlighting the potential of antiplatelet agents to attenuate NET-driven thrombosis and underscoring the need for further research to characterise the effects of these agents.

The interaction of neutrophils with platelets was first described by the term ‘thrombo-inflammation’, which nowadays is commonly used to describe the crosstalk between inflammation and thrombosis, processes where both neutrophils and NETs are critically involved [51,52]. Thrombo-inflammation is associated with a wide range of human diseases, including sepsis, COVID-19, stroke, and myocardial infarction [51], highlighting the need for further investigation on the mechanisms at play in the role of neutrophils and NETs on clot formation. Nonetheless, animal models for in vivo experiments are costly and require important ethical considerations. In vitro models using primary human neutrophils have experimental limitations, such as a large blood volume requirement from donors, short cell shelf life, and auto-activation. Therefore, neutrophil-like models may provide a viable alternative for in vitro experimental investigation of the role of neutrophil and NETs in blood clot formation.

### 1.3. Neutrophil-like PLB-985 Cells

Experimental challenges such as the short lifespan of isolated primary neutrophils and donor variability motivated the development of reproducible neutrophil-like cell models. Human myeloid cell lines have been previously used as a neutrophil-like cell model. Among them, the promyelocytic leukaemia cell line HL-60 and its sub-line PLB-985 are the most commonly used, as both can be chemically differentiated into neutrophil-like cells. Transcriptomic profiling has shown that differentiated HL-60 and PLB-985 cells share gene expression patterns closely resembling those of primary human neutrophils, making them suitable and reproducible models for neutrophil research [3,53,54,55]. Moreover, studies show that upon differentiation, PLB-985 cells acquire typical neutrophil-like morphology with segmented, multilobed nuclei and cytoplasmic granules, and exhibit key neutrophil functions such as chemotaxis, phagocytosis, and oxidative activity [3,53,54]. These cells, which can be purchased commercially, are immortalised cells that can be cultured following standard suspension cell culturing techniques. Differentiation of cells is achieved with the addition of DMSO in culturing media, leading to cellular expression of neutrophil makers (e.g., CD11b and FPR1) and phagocytic activity [56]. To add to that, we previously showed that PLB-985 cells had surface expression of CD11b comparable to isolated human neutrophils at day 6 post-differentiation (97% and 96.6% CD11b positive cells, respectively), and that CD11b expression increased with differentiation length [3].

### 1.4. Do Differentiated PLB-985 Cells Form Human-like NETs?

We, along with other researchers, have previously shown that differentiated PLB-985 cells, and their parent cell HL-60, can form NETs [3,55,57] and exhibit degranulation levels similar to those of neutrophils [54]. Nevertheless, when compared to human neutrophils, one study showed that exocytosis and phagocytosis of differentiated PLB-985 cells were weaker [53]. Whether NETs produced by PLB-985 cells resemble those formed by human neutrophil is still to be determined. Therefore, building on our recent observation that human neutrophils promote clot formation via FXI-dependent mechanisms and uniquely modulate fibrin architecture through NETs [3], we tested whether differentiated PLB-985 cells can reliably reproduce the NET-forming and procoagulant activities of human neutrophils using a combined approach of SEM and AFM imaging analyses.

## 2. Methods

The complete methodology of this study is included in the Supplementary Materials.

### 2.1. Blood Collection and Processing

Ethical approval for blood collection from healthy volunteers was granted by the University of Leeds Medicine and Health Faculty Research Ethics Committee (reference number HSLTLM12045). Blood samples were obtained from the antecubital vein with minimal stasis, and the first 2.0 mL of blood was discarded. Samples were collected into tubes containing 0.5 M Ethylenediaminetetraacetic acid (EDTA) or EDTA Vacutainers (Greiner Bio-One, Stonehouse, UK). Human neutrophils were isolated by density gradient centrifugation using Lympholyte-poly (Cedarlane, Burlington, ON, Canada), as described previously [3]. Normal pooled plasma (NPP) was prepared according to previously described protocols [58].

### 2.2. PLB-985 Cell Culture, Differentiation, and NETosis

The PLB-985 cell line (ACC-139, DSMZ, Braunschweig, Germany) was cultured and differentiated, using medium containing 1.25% DMSO, as described previously [3]. Differentiated cells were harvested for experiments on day 6. NET formation was induced by PMA and prepared for microscopy as previously described [3].

### 2.3. Scanning Electron Microscopy (SEM)

SEM was employed to visualise clot ultrastructure. Clots were formed in Eppendorf lids (with perforation holes sealed by parafilm) from mixtures containing diluted plasma (1:3), CaCl_2_ (10 mM), thrombin (1 U/mL), and either human neutrophils, differentiated PLB-985 cells, pre-generated human NETs, or pre-generated PLB-985 NETs (each at 2 × 10^6^ cells/mL). Clotting was performed for 1–2 h in a humidity chamber. Clots underwent washing (3 × 40 min in saline on a roller apparatus), fixation overnight in 2% glutaraldehyde, washing in 50 mM sodium cacodylate buffer (pH 7.4; 3 × 40 min), and progressive dehydration through acetone gradient concentrations (30–100%). After critical point drying with carbon dioxide, samples were mounted onto SEM stubs, sputter-coated with 10 nm iridium, and carbon-painted to minimise charging. Imaging was conducted using an SU8230 Ultra-High-Resolution SEM (Hitachi, Tokyo, Japan). Critical point drying and mounting were assisted by Mr. Martin Fuller (Astbury Centre, University of Leeds, Leeds, UK).

### 2.4. Atomic Force Microscopy (AFM)

Samples for AFM analysis were prepared similarly to immunofluorescence assays. After fixation and washing (3× PBS), samples were mounted onto metal plates. AFM imaging was performed in air using Tapping mode with TESPA-V2 probes (Bruker MultiMode 8 AFM, Billerica, MA, USA). Prior to air-mode imaging, samples were gently rinsed with ddH_2_O and dried under nitrogen gas. AFM images were analysed with NanoScope Analysis v1.9 (Bruker).

### 2.5. Data Analysis

AFM images were processed using NanoScope Analysis v1.9 software. Fibrin fibre diameters (80 fibres/clot, from 3–5 clots per group) and NET fibre characteristics and other components shown in SEM images (e.g., diameters of cells, filamentous structures, and spherical particles) were analysed using Fiji-ImageJ v2.1.0. Statistical analysis was conducted using GraphPad Prism 7, using the non-parametric Mann–Whitney test or the non-parametric Kruskal–Wallis test followed by Dunn’s multiple comparisons test. Statistical significance was defined as *p* < 0.05.

## 3. Results

### 3.1. SEM Confirms Neutrophil-like Morphology and Filamentous Features of PLB-985 Cells

**Figure 2:**
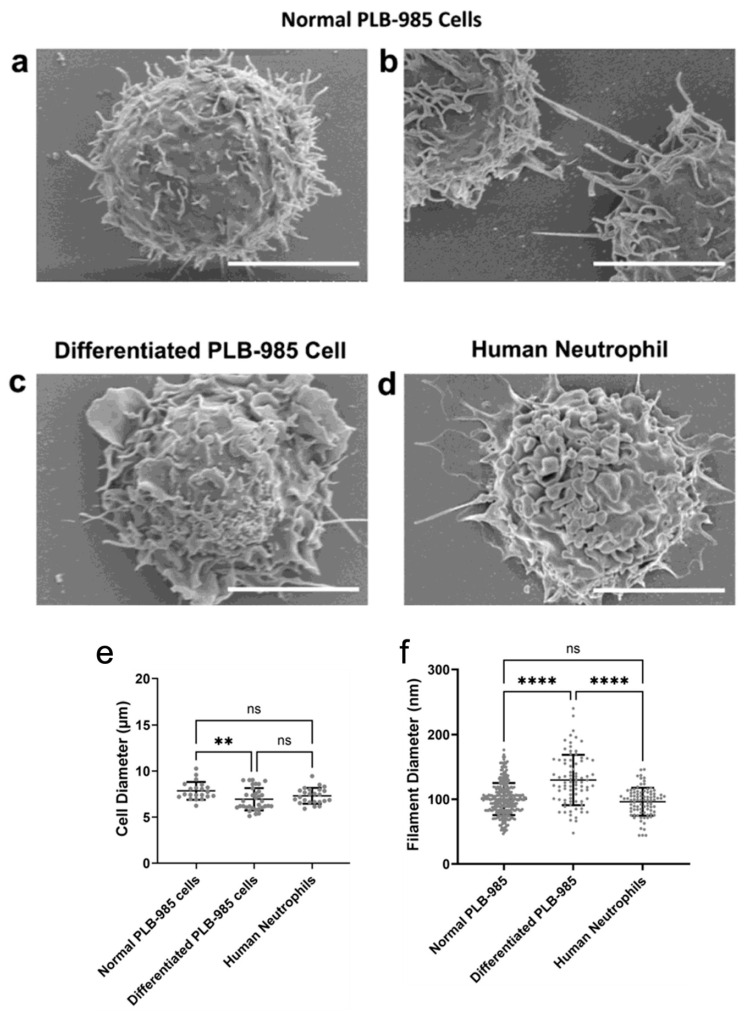
Structural analysis of differentiated PLB-985 and human neutrophil cells. SEM images of normal PLB-985 cells (**a**,**b**), a differentiated PLB-985 cell (**c**), and a human neutrophil (**d**). Scale bars are 5 µm (n ≥ 3). SEM analysis of cell diameter ((**e**), n ≥ 21) and cell surface filament diameter ((**f**), n ≥ 83). Error bars represent mean ± SD. ** *p* < 0.01, **** *p* < 0.0001, ns: non-significant. (**e**,**f**) The statistical analysis results were obtained using the non-parametric Kruskal–Wallis test followed by Dunn’s multiple comparisons test. Experiments were performed independently at least three times using separate cell preparations.

Scanning electron microscopy provided structural insight into differentiated PLB-985 cells and their similarity to human neutrophils. Differentiation induced membrane surface ruffling and flap formation in PLB-985 cells, phenotypically resembling human neutrophils (Figure 2a,d). As shown in Figure 2e, the average diameters of differentiated PLB-985 cells (6.94 µm) and human neutrophils (7.31 µm) were comparable. There was no significant difference between normal (undifferentiated) PLB-985 cells (7.85 µm) and human neutrophils. However, differentiation significantly reduced the cell diameter compared to undifferentiated PLB-985 cells (*p* < 0.01).

Filamentous structures were observed across all three cell types; in several fields, these filaments appeared to form intercellular connections (Figure 2b). There was no significant difference in average filament diameter between normal PLB-985 cells (100.3 nm) and human neutrophils (96.3 nm) (Figure 2f). In contrast, differentiated PLB-985 cells exhibited significantly thicker filaments, with an average diameter of 129.8 nm, suggesting a distinct morphological change upon differentiation.

### 3.2. AFM Shows Surface Morphology and Mechanical Properties of Differentiated PLB-985 Cells and NETs

**Figure 3:**
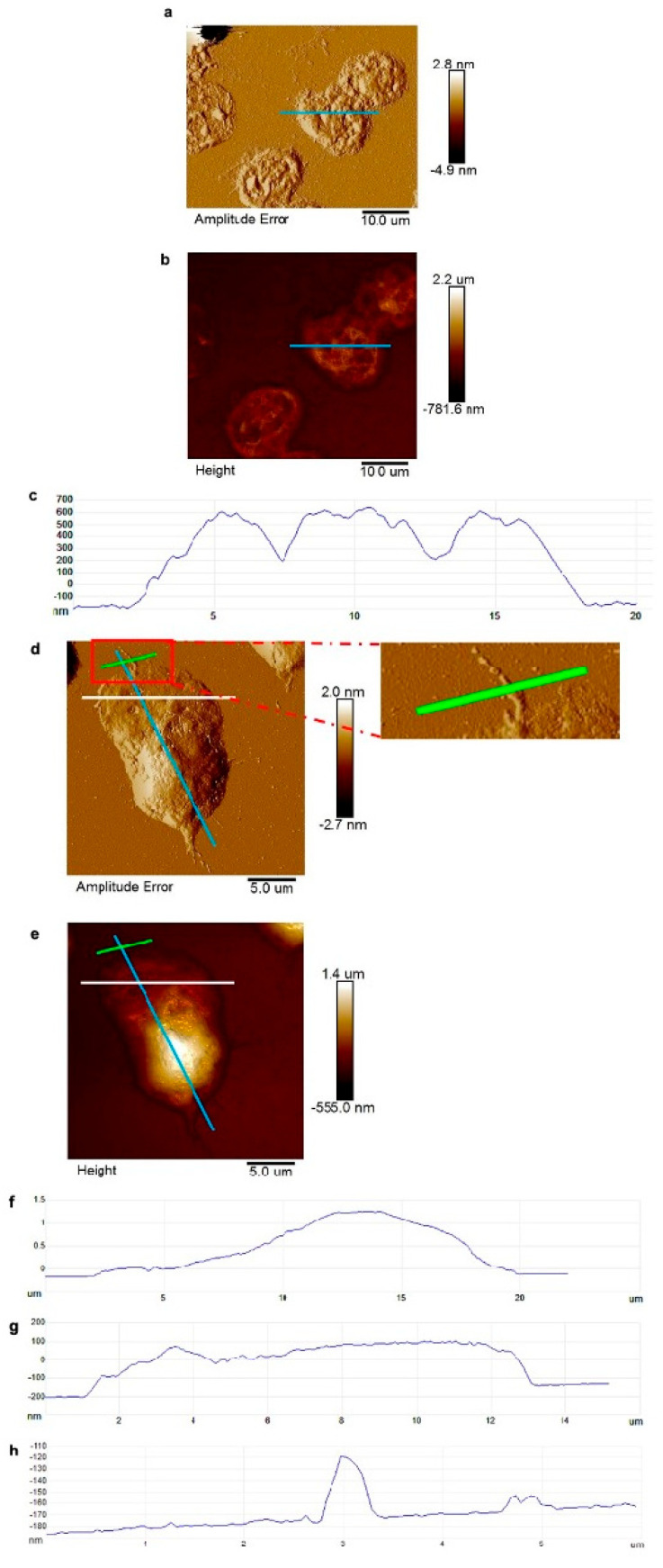
Analysis of surface morphology and mechanical properties of differentiated PLB-985 cells and their NETs. Air-dried differentiated PLB-985 cells (**a**–**c**) and air-dried PLB-985 NETs (**d**–**h**). (**a**) The amplitude error channel reflects a three-dimensional image of cells. (**b**) The height channel reflects the size range of cells. (**c**) The height profile taken along the blue line in panel. (**d**) The amplitude error channel reflects a three-dimensional image of NETs. (**e**) The height channel reflects the size range of NETs. (**f**) The height profile taken along the blue line in panel (**d**,**e**). (**g**) The height profile taken along the white line in panel (**d**,**e**). (**h**) The height profile taken along the green line in panel (**d**,**e**). Experiments were performed independently at least three times using separate cell preparations.

Neutrophil-like PLB-985 cells and their NETs were scanned in air-mode AFM. Differentiated PLB-985 cells (Figure 3a,b) exhibited a rugged membrane surface, with a mean horizontal diameter of approximately 13.5 µm and a height ranging from 378.7 to 829.0 nm (Figure 3c). AFM scanning of PLB-985-derived NETs (Figure 3d,e) revealed a central structure with an average height of 1.5 µm (Figure 3f), from which extended extracellular fibres exhibited variable heights between 5.5 and 397.8 nm (Figure 3g). Individual NET fibres imaged in high resolution had a horizontal width of approximately 0.53 µm and a height of 59.4 nm (Figure 3h). Further measurements of distinct NET fibres showed widths ranging from 0.12 to 0.20 µm and heights from 16.3 to 36.3 nm (Supplementary Materials, Figures S1–S5).

### 3.3. SEM Reveals Comparable Clot Networks Induced by Human and PLB-985 NETs

**Figure 4:**
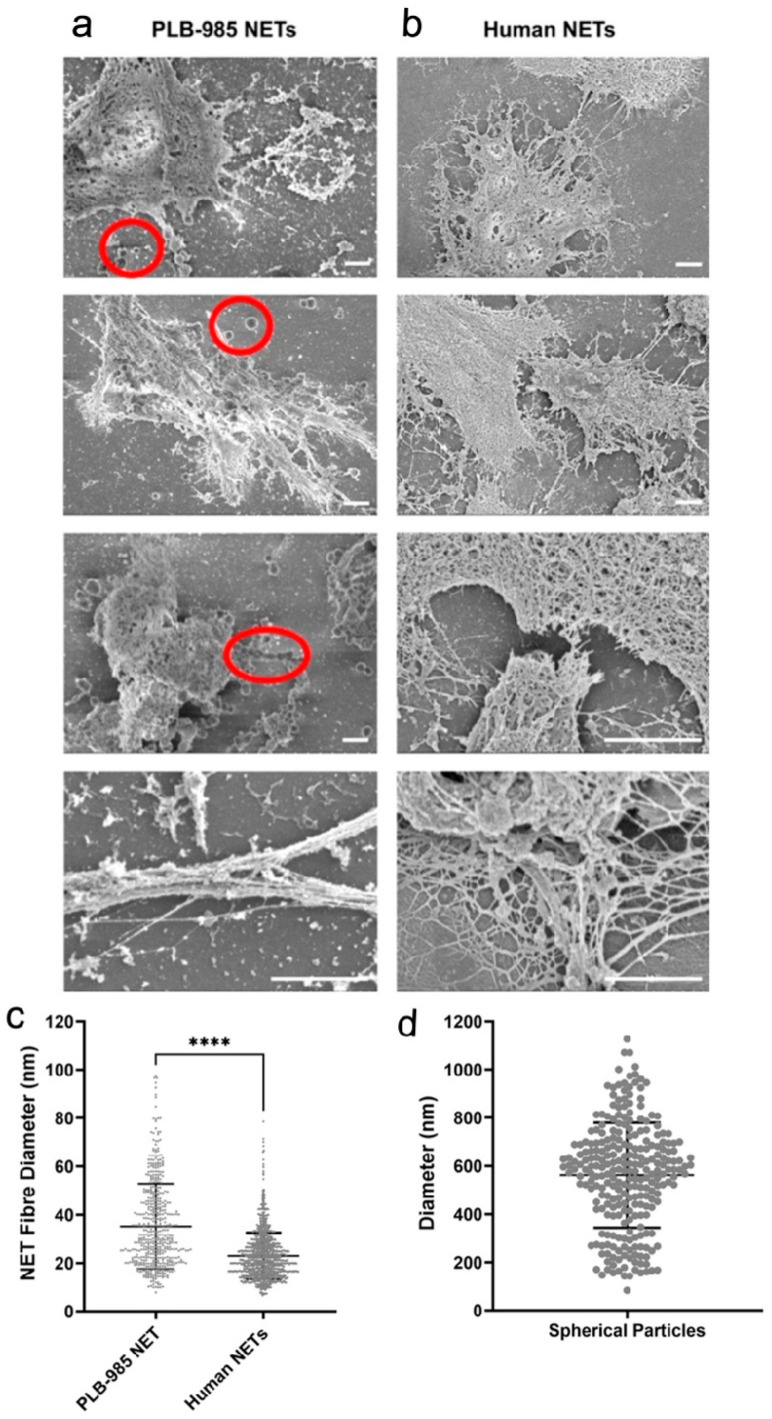
Structural analysis of differentiated PLB-985 and human neutrophil NETs. SEM images of PLB-985 NETs (**a**) and human NETs (**b**). Scale bars are 2 μm. Red circle: spherical particles, either individually or in a series (n ≥ 3). SEM analysis of NET fibre diameter ((**c**), n ≥ 514) and the diameter of spherical particles in PLB samples ((**d**), n = 261). Error bars represent mean ± SD. **** *p* < 0.0001. (**c**) The statistical analysis results were obtained using the non-parametric Mann–Whitney test. Experiments were performed independently at least three times using separate cell preparations.

To investigate the influence of NETs on fibrin architecture, clots induced by either PLB-985- or human-derived NETs were analysed using SEM. Figure 4a,b shows the presence of fine, DNA-like fibres in both PLB-985- and human-derived NETs. Single NET fibres measured approximately 7.8–97.5 nm in PLB-985 samples and 6.6–78.5 nm in human samples (Figure 4c). The average diameter of PLB-985 NET fibres (35.2 nm) was significantly greater than that of human NET fibres (23.0 nm) (*p* < 0.0001). In addition, a distinct ultrastructural feature was observed exclusively in PLB-985-derived NETs: spherical particles ranging from 84.0 to 1126.7 nm in diameter (Figure 4d, red circles in Figure 4a). These structures were not detected in NETs derived from human neutrophils.

**Figure 5:**
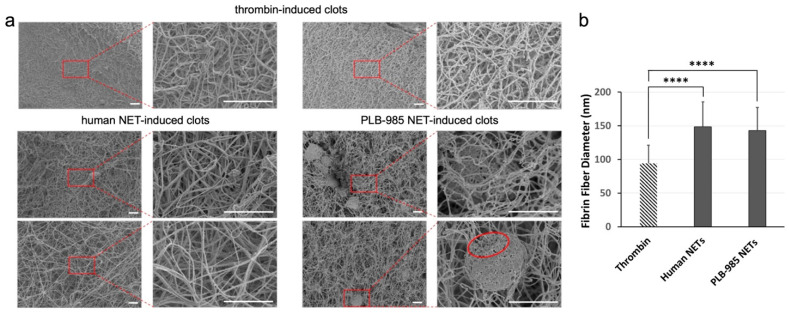
Structural analysis of clots formed with differentiated PLB-985 and human neutrophil NETs. SEM images of PLB-985 NET-induced and human NET-induced plasma clots (**a**). Thrombin-induced clots were used as a control. Final concentrations: plasma (diluted 1:3), 10 mM CaCl_2_, and 1 U/mL thrombin. Scale bars are 5 µm. Red circle: spherical particles (n ≥ 3). Effects of human NETs and PLB-985 NETs on the fibrin fibre thickness in plasma (**b**). Final concentrations: plasma (diluted 1:6), cells (200,000 cells/100 µL), NETs (generated from 200,000 cells/100 µL), thrombin (0.1 U/mL), and CaCl_2_ (3.33 mM). Error bars represent mean ± SD (n = 80). **** *p* < 0.0001, using the non-parametric Kruskal–Wallis test followed by Dunn’s multiple comparisons test. Experiments were performed independently at least three times using separate cell preparations.

SEM analysis of NET-induced clots revealed comparable overall fibrin network morphology between PLB-985 and human NET groups (Figure 5a). At higher magnification, two distinct fibre types were identifiable: long, smooth fibres with consistent thickness, likely corresponding to fibrin, and thinner, less uniform fibres consistent with NETs. These fibres were often observed to intertwine within a composite mesh. In certain images (e.g., Figure 5a, red circles), dense fibre entanglement and the integration of spherical particles suggested complex interactions between NETs and clot components. Additional SEM images are included in the Supplementary Materials (Figure S6).

### 3.4. Comparable Fibrin Fibre Diameter in NET-Induced Clots

Quantification of fibrin fibre diameter provided further insight into the procoagulant effects of NETs. Figure 5b shows that average fibrin fibre diameters in clots induced by PLB-985 NETs and human NETs did not differ significantly. Both PLB-985 and human NETs significantly increased fibrin fibre thickness compared to thrombin-only controls, by 1.52-fold and 1.58-fold, respectively (*p* < 0.0001).

## 4. Discussion

This study assessed the structural features and clot-modifying effects of differentiated PLB-985 cells and their NETs, in comparison to those derived from primary human neutrophils. Using high-resolution imaging techniques, including AFM and SEM, we demonstrated that differentiated PLB-985 cells display morphological characteristics that closely resemble mature neutrophils. These structural similarities support the suitability of PLB-985 cells as a neutrophil-like model.

Quantitative SEM data revealed that differentiated PLB-985 cells possess significantly thicker filamentous surface structures (129.8 nm) compared to undifferentiated cells and human neutrophils, indicating structural remodelling associated with differentiation. These membrane ruffling changes may reflect increased surface complexity that is potentially linked to the cells’ capacity for NET formation and features associated with neutrophil activation.

AFM analysis confirmed the ultrastructural composition of PLB-985-derived NETs, revealing NET fibre diameters ranging from 16 to 59 nm in height, which is substantially larger than the ~2 nm thickness of a single DNA helix. These findings are in agreement with prior work indicating that NET fibres are formed by multiple strands of decondensed chromatin and histone complexes [59,60]. SEM images further supported this architecture, showing extensive NET networks in both PLB-985 and human samples. Notably, NETs formed by PLB-985 cells exhibited thicker fibres on average than those from human neutrophils (35.2 nm vs. 23.0 nm), suggesting potential differences in chromatin density or NET-associated protein composition. Whether these differences impact the mechanical properties or biological activity of the NETs remains to be clarified.

A striking and exclusive feature observed in PLB-985-derived NETs was the presence of spherical structures (84–1127 nm) that are morphologically consistent with microvesicles. Although the nature of these structures has not been identified, these particles may reflect tissue factor (TF)-bearing extracellular vesicles, which are known to contribute to thrombin generation and coagulation in cancer-derived and myeloid cell lines [61,62,63]. The presence of these spherical particles resembling vesicles in PLB-985 NETs indicates a potential additional procoagulant mechanism distinct from NET-mediated fibrin modification. However, further studies are required to confirm the identity and functionality of these vesicle-resembling particles in clot formation.

In our previous study, PLB-985 cells were identified as a potential substitute for neutrophils in NET research, albeit with certain caveats and alongside confirmation of key findings using primary neutrophils [3]. The present work advances this by examining in detail the ultrastructure of both primary neutrophil-derived and PLB-985-derived NETs and their direct impact on fibrin architecture. One important consideration is that cellular heterogeneity directly affects NET formation, which in turn may influence downstream clot structure. Therefore, it is crucial to ensure effective cellular differentiation of neutrophil-like cell models through measures of neutrophil surface marker expression, for instance [3]. Our findings consistently reinforce the procoagulant effects of NETs, derived from both PLB-985 cells and primary neutrophils, to modify clot structure, particularly through increasing fibrin fibre thickness. This structural modification is consistent with earlier reports suggesting that NET-associated molecules, primarily DNA and histones, enhance fibrin polymerisation, thus altering clot morphology and stability [64,65]. Further studies investigating the effects of DNA degradation and histone-mediated chromatin decompensation are required to identify the potential roles of intact NETs and/or cellular-derived components on fibrin structure.

A notable challenge identified in our SEM studies was the indistinguishable morphology between NET fibres and fibrin fibres. These two fibre types often appear intertwined and indistinguishable in electron micrographs, complicating the analysis of their respective contributions to clot structure. In this context, we propose that future studies could exploit combined AFM–fluorescence imaging, which is a powerful, emerging technique enabling simultaneous high-resolution morphological characterisation and fluorescently labelled molecular identification [66]. Additionally, in-liquid AFM allows for high-resolution imaging of fibre topography under physiological conditions without extensive sample preparation, thus preserving the native structural integrity of biological samples. Such an approach would facilitate precise differentiation of fibrin and NET fibres, providing novel insights into their specific interactions and distinct roles in thrombus formation.

In summary, our results demonstrate that PLB-985-derived NETs share key morphological and functional features with human neutrophil NETs. Both promote clot formation by thickening fibrin fibres and generating comparable clot architectures. These findings validate the differentiated PLB-985 cell line as an ethically responsible, practical, and reproducible alternative to primary human neutrophils for in vitro thrombosis studies, and extend the findings of our prior work [3] by providing detailed structural validation of this model. Researchers should, nevertheless, remain mindful of differences in granular content, signalling pathways, and NET composition when using PLB-985 and other neutrophil-like cells as replacements for primary cells. Further research into the molecular composition of PLB-associated vesicles and the identification of functional procoagulant markers will provide additional clarity on the precise mechanisms by which these cells influence coagulation. NETs play important clinical roles by contributing to host protection during wound healing by defending against microbial infections [1,67]. They are also implicated in both venous and arterial thrombosis, including venous thromboembolism and stroke, where they have been detected within thrombi and associated with resistance to thrombolytic therapy [31,32,33]. In this context, our review and research on PLB-985 cells are highly relevant, as these cells can serve as in vitro models for studying neutrophils and NET formation under specific conditions, provided that key findings are validated using primary neutrophils.

## Data Availability

The raw data supporting the conclusions of this article will be made available by the authors upon request.

## Supplementary Materials

The following supporting information can be downloaded at: https://www.mdpi.com/article/10.3390/cells15060541/s1.
