# Supplementary material for: A Neutrophil-like Cell Model as Substitute for Human Neutrophils in NETs and Thrombosis Research
Source: Cells. 2026 Mar 18;15(6):541. doi: 10.3390/cells15060541 (PMC13024864; doi:10.3390/cells15060541)
Supplement: Supplementary file 1 [file cells-15-00541-s001.zip › cells-4151069-supplementary.pdf]

A Neutrophil-like Cell Model as Substitute for Human Neutrophils in NETs and Thrombosis Research

Yu Shi, Helen R. McPherson, Timea Feller, Simon D. A. Connell, Helen Philippou, Robert A. S. Ariëns and Julia S. Gauer

Supplementary Information

METHODS IN FULL

Ethical approval and blood sample collection

All blood samples were collected following informed written consent from healthy donors in accordance with the Declaration of Helsinki. Ethical approval was granted by the University of Leeds Medicine and Health Faculty Research Ethics Committee (reference number HSLTLM12045). Blood samples were obtained from the antecubital vein with minimal stasis, and the first 2.0 ml of blood was discarded. Samples were collected into tubes containing 0.5 M Ethylenediaminetetraacetic acid (EDTA) or EDTA Vacutainers (Greiner Bio-One, Stonehouse, UK), as previously described[1].

Isolation of human neutrophils

Human neutrophils were isolated by density gradient centrifugation using Lympholyte-poly (Cedarlane, Burlington, ON, Canada), as described previously[1]. Briefly, 5 ml of Lympholyte-poly was carefully layered on top of 5 ml whole blood in a 15 ml Falcon tube and centrifuged at 500 RCF for 35 min at 23°C without brakes. Following centrifugation, six distinct layers formed: plasma, mononuclear cells, isolation media, polymorphonuclear cells (neutrophils), isolation media, and red blood cells (from top to bottom). If layer separation was unclear, centrifugation was repeated for an additional 15–20 min. The upper three layers were discarded, and the neutrophil-containing layer along with underlying media was carefully transferred to a new Falcon tube. Cells were washed with 10 ml Hank's Balanced Salt Solution (HBSS) without Ca^{2+}/Mg^{2+} (Gibco, London, UK) and centrifuged (350 RCF, 10 min). Residual red blood cells were lysed with 2 ml Red Blood Cell Lysis Buffer (Roche, Hertfordshire, UK). Following gentle resuspension, cells were washed again (HBSS without Ca^{2+}/Mg^{2+}, 250 RCF, 5 min). Lysis was repeated as required. The final cell pellet was resuspended in HBSS (with Ca^{2+}/Mg^{2+}) or HEPES-buffered saline (HBS), supplemented with either 2% w/v human serum albumin (HSA) or 2% v/v fetal bovine serum (FBS), depending on downstream assays. Viable neutrophil numbers were determined using Trypan Blue staining and counted on a haemocytometer under an Olympus CKX41 inverted microscope (Tokyo, Japan).

Normal pooled plasma preparation

Normal pooled plasma (NPP) was prepared according to previously described protocols[2]. Blood was collected from the antecubital vein of at least 25 healthy donors using 19-gauge butterfly needles into tubes containing 0.109 M trisodium citrate. Blood samples were centrifuged at 3,000 RCF for 20–30 min within one hour of collection to obtain platelet-poor plasma, which was then pooled, aliquoted into 0.8–1 ml volumes, snap-frozen in liquid nitrogen, and stored at –80°C. Prior to use, plasma aliquots were thawed at ~37ºC for 5 min, centrifuged at 10,000 RCF for 10 min at room temperature, and filtered through 0.2 µm filters.

PLB-985 cell culture and differentiation

The PLB-985 cell line (ACC-139, DSMZ, Braunschweig, Germany), a human acute myeloid leukemia cell line, was cultured and differentiated as described previously[1]. PLB-985 cells were cultured in RPMI 1640 medium (R8758, Sigma-Aldrich, Suffolk, UK) supplemented with 10% heat-inactivated FBS at 37°C in a humidified incubator with 5% CO_2. Cells were routinely passaged every two days. For long-term storage, PLB-985 cells (1×10^6 cells/ml) were cryopreserved in liquid nitrogen using freezing medium (70% RPMI 1640, 20% FBS, 10% dimethyl sulfoxide [DMSO; Sigma-Aldrich, Suffolk, UK]). For differentiation into neutrophil-like cells, cells were cultured for 5 days in RPMI 1640 medium containing 1.25% DMSO and 5% heat-inactivated FBS, with a medium refresh on day 3. Differentiated cells were harvested for experiments on day 6.

Generation of NETs for microscopies analysis

NET formation was induced and prepared for microscopy as previously described [40]. NET formation assays were conducted in 24-well plates containing poly-L-lysine-coated round coverslips. Isolated human neutrophils (2×10^5 cells/well) were seeded in 500 µl HBSS supplemented with Ca^{2+}/Mg^{2+} and 2% HSA. Differentiated PLB-985 cells (2×10^5 cells/well) were seeded in RPMI 1640 medium supplemented with 2% FBS. Cells were allowed to adhere for 1 hour at 37°C, after which media were carefully removed and replaced with 20 or 100 nM phorbol 12-myristate 13-acetate (PMA, Sigma-Aldrich, Suffolk, UK) diluted in respective media. Cells were incubated at 37°C for 2 hours or overnight to allow for NET formation.

REFERENCES

1. Shi Y, Gauer JS, Baker SR, Philippou H, Connell SD, Ariëns RAS. Neutrophils can promote clotting via FXI and impact clot structure via neutrophil extracellular traps in a distinctive manner in vitro. Sci Rep. 2021;11:1718.

2. Macrae FL, Duval C, Papareddy P, Baker SR, Yuldasheva N, Kearney KJ, McPherson HR, Asquith N, Konings J, Casini A, Degen JL, Connell SD, Philippou H, Wolberg AS, Herwald H,

Ariens RA. A fibrin biofilm covers blood clots and protects from microbial invasion. J Clin Invest. 2018;128:3356-3368.

SUPPLEMENTARY FIGURES

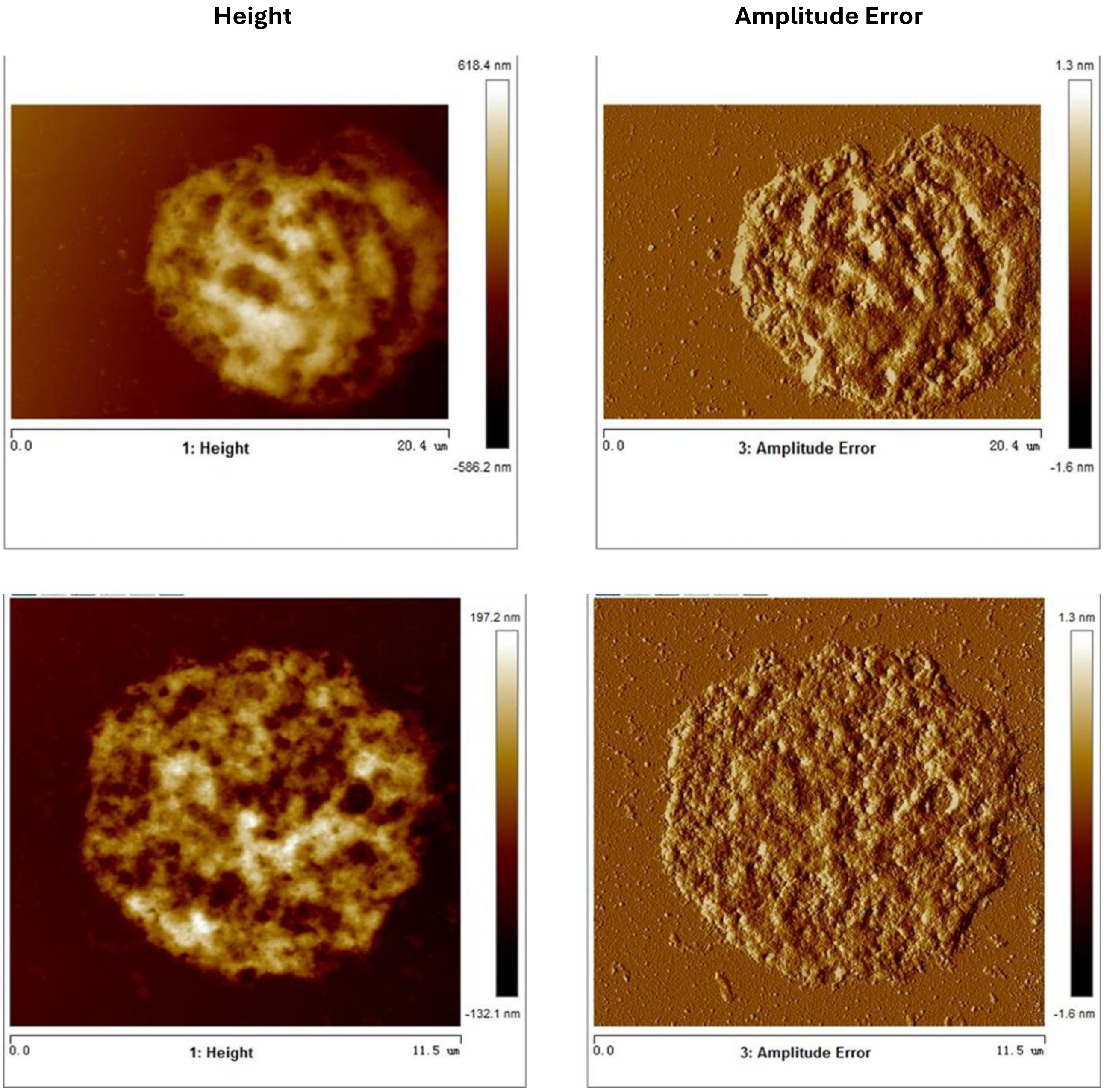

Figure S1. Air-dried PLB-985 Cells. Height channel reflects the size range of cells. Amplitude error channel reflects a three-dimensional image of cells.

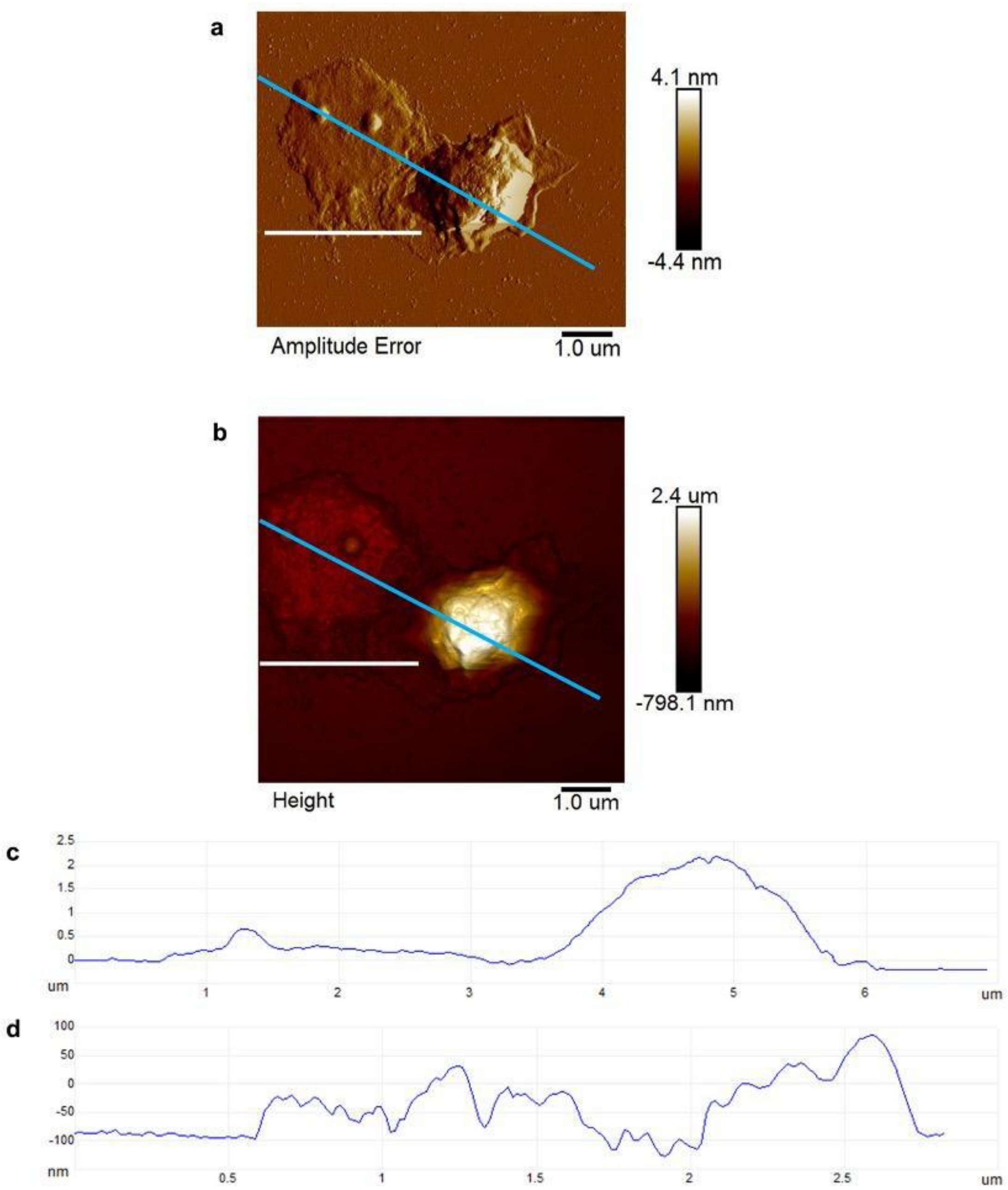

Figure S2. AFM analyses of air-dried PLB-985 NETs. (**a**) Amplitude error channel reflects a three-dimensional image of NETs. (**b**) Height channel reflects the size range of NETs. **(c)** Height profile taken along the blue line in panel **a** and **b**. (**d**) Height profile taken along the white line in panel **a** and **b**.

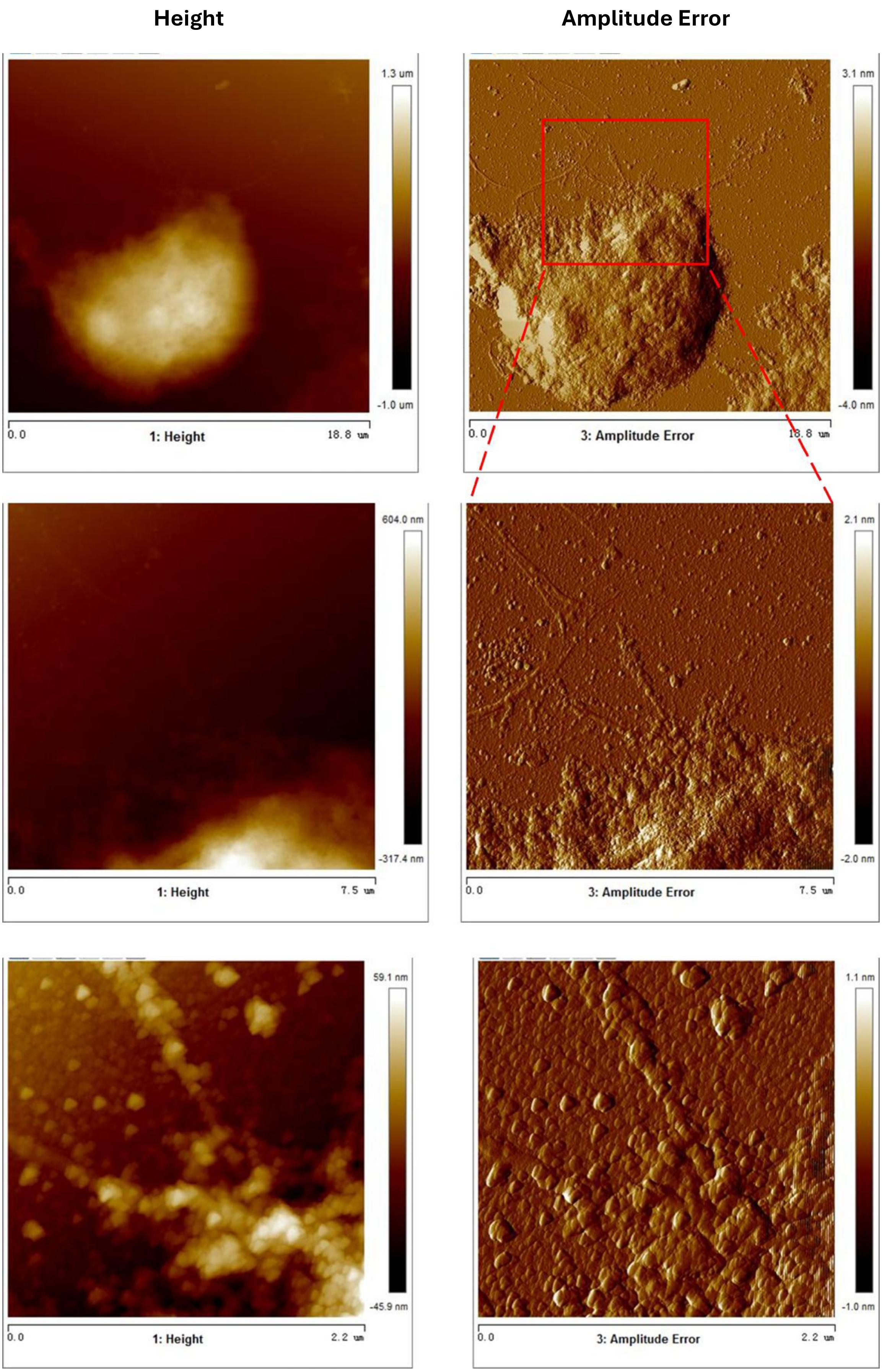

Figure S3. Air-dried PLB-985 NETs. Height channel reflects the size range of NETs. Amplitude error channel reflects a three-dimensional image of NETs.

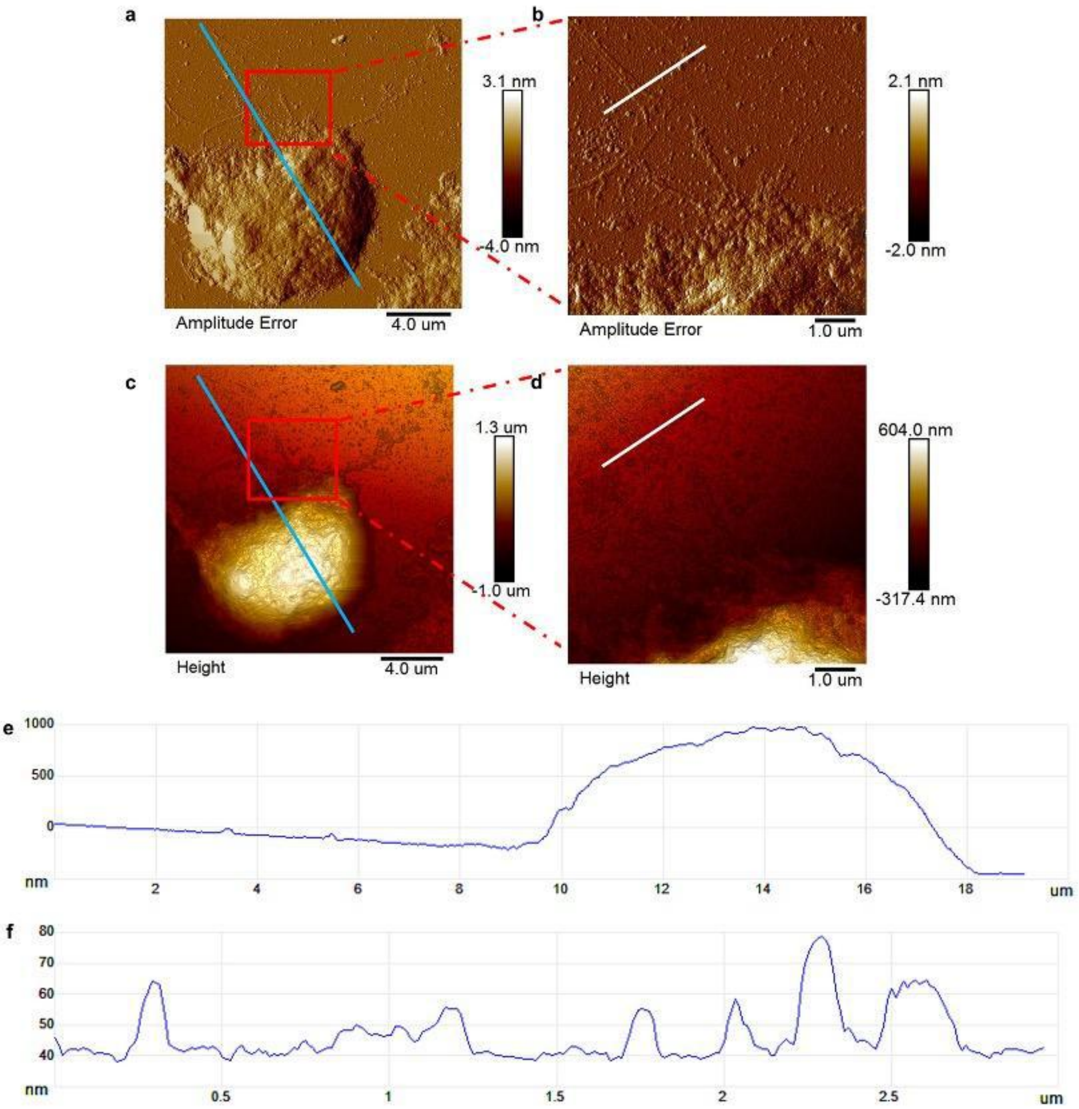

Figure S4. AFM analyses of air-dried PLB-985 NETs. (**a**) Amplitude error channel reflects a three-dimensional image of NETs. (**b**) Height channel reflects the size range of NETs. **(e)** Height profile taken along the blue line in panel **a** and **c**. (**f**) Height profile taken along the white line in panel **b** and **d**.

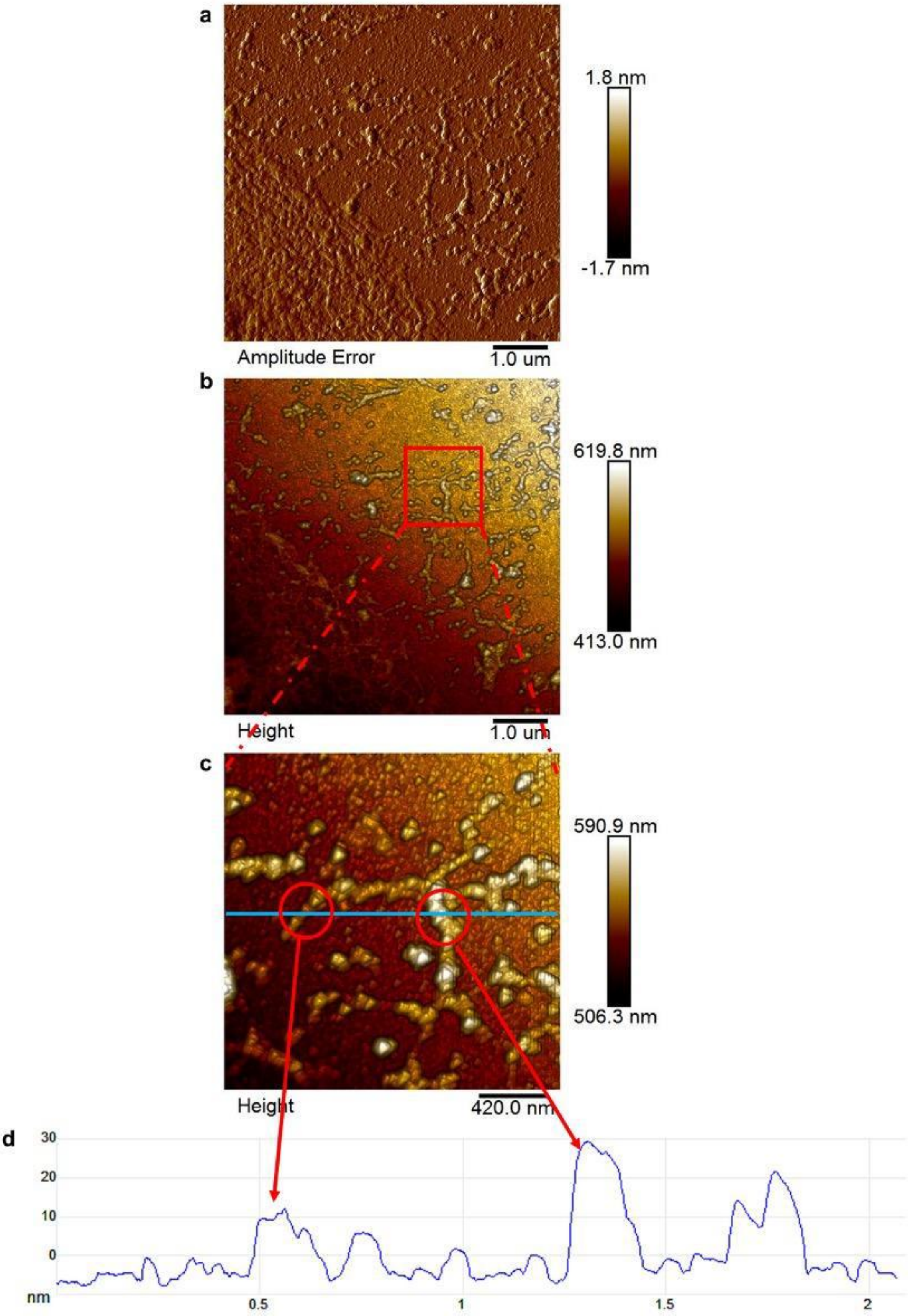

Figure S5. AFM analyses of the single fibres of air-dried PLB-985 NETs. (**a**) Amplitude error channel reflects a three-dimensional image of NETs. (**b** and **c**) Height channel reflects the size range of NETs. (**d**) Height profile taken along the blue line in panel (**c**).

Thrombin-induced Clots

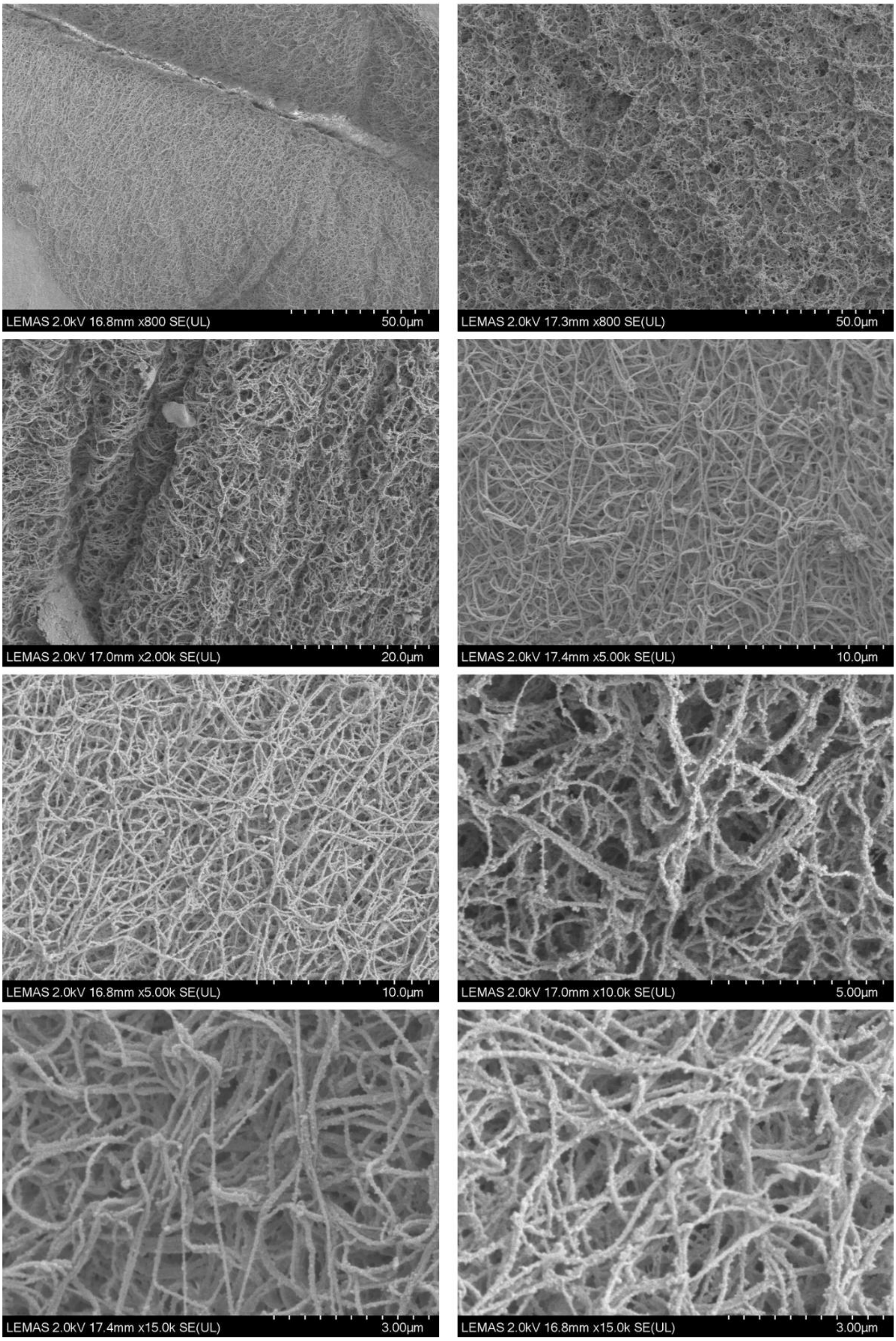

PLB-985 NET-induced Clots

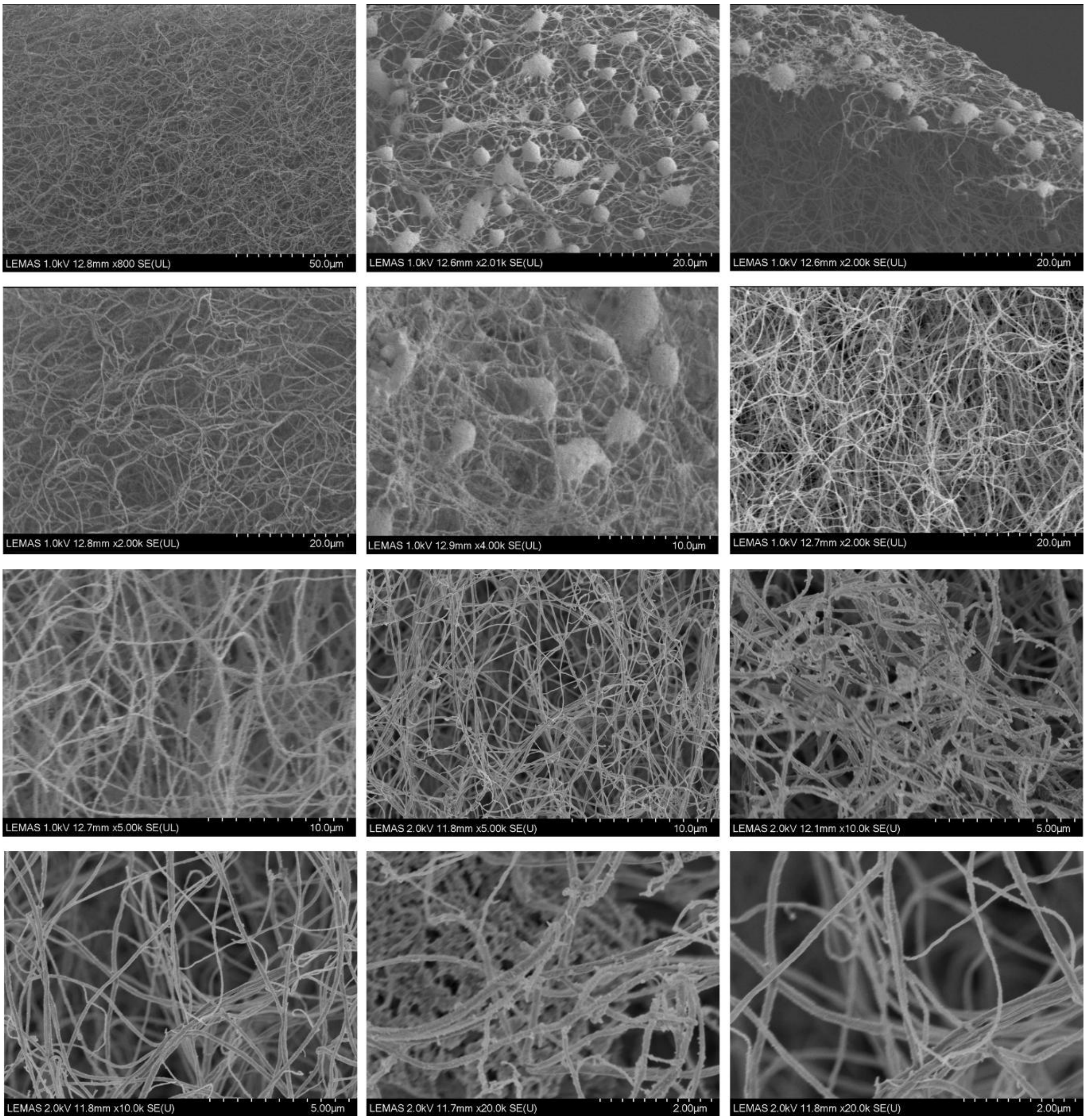

Human NET-induced Clots

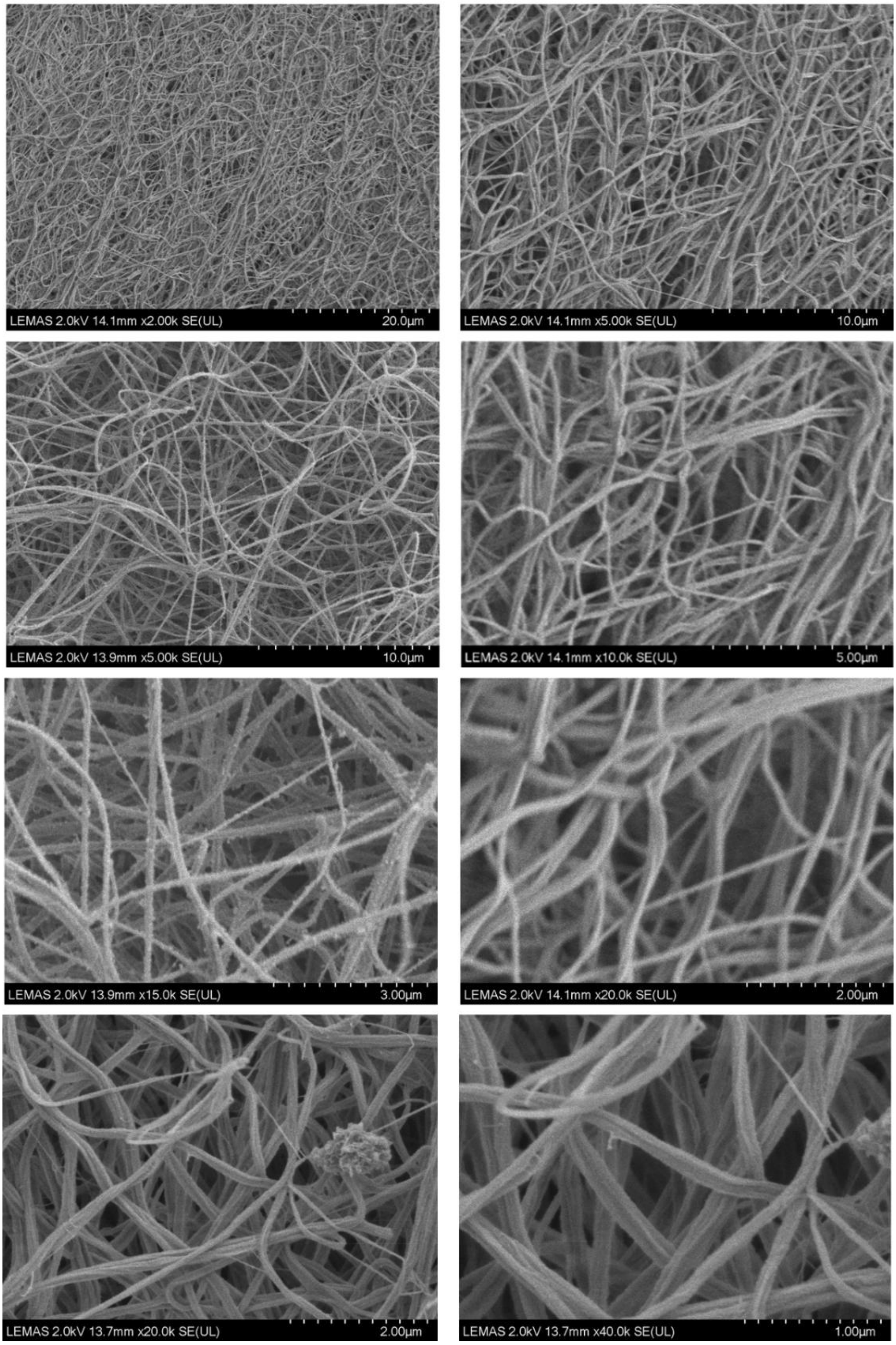

Figure S6. Additional SEM images of clots induced by PLB-985 NETs and human NETs. Thrombin-induced clots were used as a control. Final concentrations: Plasma (diluted 1:3), 10 mM CaCl2, 1 U/ml thrombin. Some images were used in the fibrin fibre thickness analysis.
